# Morphological evidences indicate that the interference of cimetidine on the peritubular components is responsible for detachment and apoptosis of Sertoli cells

**DOI:** 10.1186/1477-7827-6-18

**Published:** 2008-05-09

**Authors:** Estela Sasso-Cerri, Paulo S Cerri

**Affiliations:** 1 Laboratory of Histology and Embryology – Department of Morphology, Dental School of São Paulo State University (UNESP), Araraquara, São Paulo, Brazil

## Abstract

Cimetidine, referred as antiandrogenic agent, has caused alterations in the seminiferous tubules, including alterations in the peritubular tissue and death of myoid cells by apoptosis. Regarding the structural and functional importance of the peritubular tissue for the maintenance of Sertoli cells (SC), we purpose to investigate the SC-basement membrane interface, focusing the morphological features of SC and their interaction with the basement membrane in the affected tubules by cimetidine. Ten animals were distributed into two groups, control (CG) and cimetidine (CmG) which received saline solution and 50 mg of cimetidine per kg of body weight, respectively, for 52 days. The testes were fixed, dehydrated and embedded for analyses under light and transmission electron microscopy. Paraffin sections were submitted to the TUNEL method; sections of testes embedded in glycol methacrylate were submitted to PAS method and stained by H&E for morphological and quantitative analyses of Sertoli Cells. In the CmG, the SC nuclei were positive to the TUNEL method and showed typical morphological alterations of cell death by apoptosis (from early to advanced stages). A significant reduction in the number of Sertoli Cells was probably due to death of these cells by apoptosis. A close relationship between SC nuclear alterations (including a high frequency of dislocated nuclei from the basal portion) and damage in the peritubular tissue was observed. The ultrastructural analysis showed a parallelism between the gradual advancement of apoptotic process in SC and detachment of the anchoring sites (hemidesmosomes) of SC plasma membrane from the lamina densa. The presence of portions of lamina densa underlying the detached hemidesmosomes indicates a continuous deposition of lamina densa, resulting in the thickening of the basal lamina. The results indicate a possible disarrangement of the SC cytoskeleton, including the focal adhesion structure. These alterations are related to SC apoptosis and probably result from disturbs induced by cimetidine on the peritubular tissue.

## Background

Cimetidine, an H_2 _receptor antagonist, is used for the treatment of gastric and duodenal ulcers and has been referred as an antiandrogenic agent in regards to its competitive action by androgen receptors [1]. In the cimetidine-treated rats, several morphological changes have been demonstrated in the seminiferous tubules [2-5]. Among them, the presence of sloughed germ cells in the lumen, loss of contact between spermatids and Sertoli cells (SC), multinucleated giant cells derived from round spermatids and germ cell loss by apoptosis have suggested an interference of cimetidine on the histoarchitecture of the seminiferous epithelium [4,5]. A significant reduction of peritubular tissue and loss of peritubular myoid cells by apoptosis, evidenced by TUNEL [3,5] and transmission electron microscopy [3] have been demonstrated in cimetidine-treated rats. Additionally to the contractile function of myoid cells, these cells seem to secrete factors that modulate SC activity [6] and express androgen receptors whose absence leads to the impairment of the SC junctions [7].

Depletion of germ cells has been associated to SC injury in rats treated with chemical compounds such as mono-(2-ethylhexyl)phthalate [8], cisplatin [9] and indenopyridine – CDB-4022 [10]. The structural integrity of the seminiferous epithelium is maintained by the developed SC cytoskeleton. The intermediate filaments, such as vimentin filaments, are concentrated around the SC nuclei, extending outward toward the lumen and associated with desmosomes and hemidesmosomes junctional complexes which maintain cell-cell and cell-matrix adhesion, respectively [11,12]. The loss of germ cells by apoptosis in the seminiferous epithelium, caused by cryptorchidism [12,13], by treatment with chemical compounds [8] and by decreased intratesticular testosterone [14], have been associated with disturbances in the vimentin filaments of the Sertoli Cells. The loss of germ cells by apoptosis after cimetidine treatment has been associated to a possible occurrence of SC death, evidenced by the TUNEL method [5]. Some findings concerning apoptosis in Sertoli Cells has been described "in vitro" [15,16]. However, to our knowledge, typical features of SC undergoing cell death by apoptosis "in vivo" have not been demonstrated. Regarding the effects of cimetidine on the peritubular tissue, affecting peribular myoid cells, we purpose to evaluate, under light and electron microscopy, the morphological features of the Sertoli Cells adjacent to the peritubular tissue affected by cimetidine. We also investigated a possible relation between the gradual alterations in the Sertoli cells and the changes in the adjacent basement membrane.

## Methods

### Animals and treatment

Adult Holtzman male rats (*Rattus norvegicus albinus*) aging 80-day-old (250–300 g) were maintained at 25°C, standard lighting conditions (12-h light/dark cycle), fed laboratory rat chow and given water *ad libitum*. Principles of laboratory animal care and national laws on animal use were observed. The protocol of this study was authorised by Ethical Committee for Animal Research of the Dental School of São Paulo State University (UNESP-Araraquara).

Two groups (control and cimetidine) containing five animals each were utilized. The animals from the cimetidine group (CmG) received injections containing aqueous solution of 50 mg of cimetidine (Tagamet^®^, SmithKline Beecham, Brazil) per kg of body weight. This dosage was selected based on the dosages used in humans that vary from 400 mg/kg/day (for treatment of hyperacidity) to 800 mg/kg/day (for treatment of acute ulcer). These dosages are equivalent, in rats, of about 35 mg/kg/day and 70 mg/kg/day, respectively. Thus, we have used the intermediate dose of 50 mg/kg [4,5]. Clinically, cimetidine can be administered via oral or intravenous; in this study, we treated the rats with intraperitoneal injections – the most adequate via of administration recommended for rodents. The animals from control group (CG) received saline solution by the same route. Since, in general, the clinical use of cimetidine in humans is over a long duration, the rats received the treatment for 52 days; moreover, this period also corresponds to the duration of spermatogenesis in adult rats [17].

### Light microscopy

The animals were anaesthetised and sacrificed with chloral hydrate and the testes were fixed in 4% formaldehyde (freshly prepared from paraformaldehyde) buffered at pH 7.2 with 0.1 M sodium phosphate for 48 hours at room temperature. Subsequently, the specimens were dehydrated in graded ethanol and embedded in glycol methacrylate (Historesin Embedding kit, JUNG, Germany). The sections were stained with haematoxylin and eosin (H&E) and submitted to Periodic Acid-Schiff (PAS) method, according to Cerri and Sasso-Cerri [18], for morphological and quantitative analyses.

Some testicular fragments were dehydrated in graded concentrations of ethanol and embedded in paraffin for detection of cell death by the TUNEL method.

#### Quantitative and statistical analyses of Sertoli cells

With the aim to verify a possible interference of cimetidine in the number of Sertoli Cells, SC nuclei showing evident nucleolus were quantified in cross seminiferous tubule sections stained by H&E [19]. For each animal, three non-serial testicular sections were used and, in each section, ten tubules were quantified totalizing 30 tubules per animal. During this analysis, the number of dislocated Sertoli cell nuclei from the basal compartment to the adluminal and/or luminal portions was also computed. The results were statistically analysed using the software Jandel SigmaStat 2.0. The one-way ANOVA followed by the Mann-Whitney test was used to compare differences between groups. Significance was accepted at a confidence level of p ≤ 0.05.

#### TUNEL method

The TUNEL (Terminal deoxynucleotidyl-transferase-mediated dUTP Nick End Labelling) method was performed as previously described [5] and according to the kit Apop-Tag Plus (Chemicon Internacional, USA). Thus, the sections adhered to silanized slides (3-aminopropyltrithoxysylane – Sigma-Aldrich Chemical Co., St. Louis, USA) were treated with 20 μg/ml proteinase K (Sigma-Aldrich Chemical Co., St. Louis, USA) and immersed in 3% hydrogen peroxide. After immersion in equilibration buffer for 20 min, the sections were incubated in TdT enzyme (Terminal deoxynucleotidyl Transferase) at 37°C for 1 hour in a humidified chamber. The reaction was stopped by immersion in a stop/wash buffer for 20 min and incubated in anti-digoxigenin-peroxidase in humidified chamber at 37°C for 30 min. The reaction was revealed with 0.06% 3.3'-diaminobenzidine tetrahydrochloride (DAB – Sigma-Aldrich Chemical Co., St. Louis, USA) and counterstained with Carazi's haematoxylin. Sections of involuting mammary gland, provided by the manufacturer of the Kit, were used as positive controls for the TUNEL method. Negative controls were incubated in a TdT enzyme-free solution.

### Transmission electron microscopy (TEM)

The specimens were fixed in a mixture of 4% glutaraldehyde and 4% formaldehyde (freshly prepared from paraformaldehyde) buffered at pH 7.2 with 0.1 M sodium cacodylate at room temperature for 16–20 hours. After washings in 0.1 M sodium cacodylate at pH 7.2, the specimens were transferred to cacodylate-buffered 1% osmium tetroxide at pH 7.2 for 1 hour at room temperature. Then, the specimens were treated with 0.5% uranyl acetate for 2 hours. After dehydration in graded concentrations of ethanol, the specimens were treated with propylene oxide and embedded in Araldite.

Semithin sections stained with 1% toluidine blue were examined in a light microscope, and suitable regions were carefully selected for trimming of the blocks. Ultrathin sections were collected on collodion-coated grids, stained in alcoholic 2% uranyl acetate and lead citrate and examined in a transmission electron microscope (Philips-CM 200).

## Results

### Light microscopy

**Figure 1:**
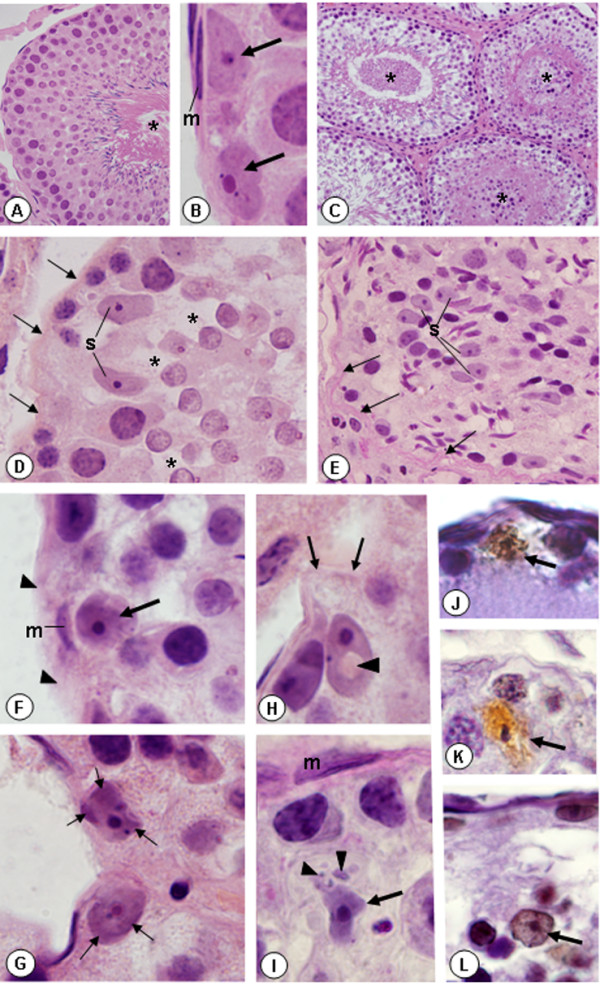
**Photomicrographs of seminiferous tubules of rats from CG (**A **and **B**) and CmG (**C**-**L**) stained by H&E (**A-H**), PAS (**I**) and submitted to TUNEL method (**J-L**). **In **A**, the tubule shows organized histoarchitecture and absence of germ cells detached in the tubular lumen (asterisk). ×400. In **B**, Sertoli cell nuclei with evident nucleolus (arrows) are juxtaposed to the peritubular tissue exhibiting myoid cell with normal aspect (m). ×1,500. In **C**, the damaged tubules show intraepithelial spaces and germ cells filling the tubular lumen (asterisks). ×170. In **D **and **E**, the peritubular tissue is irregularly outlined (arrows) and the SC nuclei (S) are dislocated from the basal portion (Fig. **D**) or positioned in the adluminal compartment (Fig. **E**). In **D**, spaces between round spermatids and Sertoli cells (asterisks) are observed. Fig. D: ×800; Fig. E: ×600. In **F**, the peritubular tissue is not evident (arrowheads) and a myoid cell nucleus shows abnormal aspect (m). The adjacent SC nucleus exhibits intense basophilic staining (arrow). In **G**, two SC nuclei with peripheral chromatin strongly stained by hematoxylin (arrows). In **H**, a portion of the peritubular tissue is not evident (arrows); the SC nucleus dislocated from the peritubular tissue shows a central clear halo (arrowhead). In **I**, an abnormal myoid cell nucleus (m) and a SC nucleus with irregular shape (arrow) is located in the adluminal compartment. Next to the SC nucleus, basophilic structures (probably nuclear fragments) are observed (arrowheads). Figs. F-I: ×1,500. In **J**, **K **and **L**, the SC nuclei are positive to the TUNEL method (arrows) and some of them (**K **and **L**) are dislocated from the peritubular tissue. Figs. J-L: ×1,400.

The seminiferous tubules of animals from CG showed normal histoarchitecture. The germ cells were organized in concentric layers and detached germ cells in the tubular lumen were rarely found (Fig. 1A). The SC nuclei were always found in the basal portion of the tubules, adjacent to the peritubular tissue. These triangular/ovoid nuclei were usually slightly stained by haematoxylin and showed a conspicuous nucleolus (Fig. 1B). In the CmG, the damaged seminiferous tubules showed irregular shape, intraepithelial spaces and detached germ cells filling the tubular lumen (Fig. 1C). Intraepithelial spaces between elongating spermatids and Sertoli Cells were usually observed. Portions of the peritubular tissue including myoid cells were not evident and the SC nuclei were dislocated from the basal compartment. In some tubules, the peritubular tissue was irregularly outlined and the elongate spermatids were abnormally distributed in the epithelium. In these tubules, SC nuclei were found in the adluminal compartment intermingle with the disarranged spermatids (Figs. 1D and 1E). In several seminiferous tubules, nuclei of SC exhibiting condensed peripheral chromatin strongly stained by haematoxylin were observed (Figs. 1F and 1G). In some nuclei, a conspicuous clear halo was found and, occasionally, basophilic bodies similar to the nucleus (probably nuclear fragments) were found next to irregular SC nucleus (Figs. 1H and 1I). Adjacent to these altered SC nuclei, the peritubular tissue was not evident and/or the myoid cell nucleus exhibited abnormal aspect (Figs. 1F–1I). In the sections submitted to TUNEL method, additionally to the TUNEL-positive germ cells, TUNEL-positive SC nuclei with evident nucleolus were found and some of them were dislocated from the basal portion (Figs. 1J–1L). The mammary gland sections (positive controls) showed numerous TUNEL-positive nuclei; in the testicular sections, used as negative controls, TUNEL positivity was not observed (data not illustrated).

### Number of Sertoli cell nuclei

According to table 1, the total number of Sertoli Cells decreased in all animals from the CmG in comparison to the CG. In the CmG, the number of Sertoli Cells reduced 18.5% and a high frequency of dislocated Sertoli cell nuclei was observed in all animals of CmG. The differences between the groups were statistically significant (p ≤ 0.05).

**Table 1:** Number of Sertoli cells (SC) and frequency of dislocated Sertoli cell nuclei (DSN) in the testes of rats from Control (C) and Cimetidine (Cm) groups

**Animal Group**	**Total SC**	**SC/Tubule**	**DSN (%)**
C1	328	10.9	1.2
C2	358	11.9	0
C3	319	10.6	0.3
C4	320	10.6	2.2
C5	387	12.9	0.5
**Mean**	**342 ± 29.53**	**11.4 ± 1.00**	**0,84 ± 0.87**
Cm1	275	9.1	18.2
Cm2	268	8.9	17
Cm3	276	9.2	11.2
Cm4	280	9.3	17.5
Cm5	297	9.9	13.8
**Mean**	279 ± 10.84*	9.3 ± 0.37*	15.5 ± 2.95*

*p ≤ 0.05

### Transmission electron microscopy

**Figure 2:**
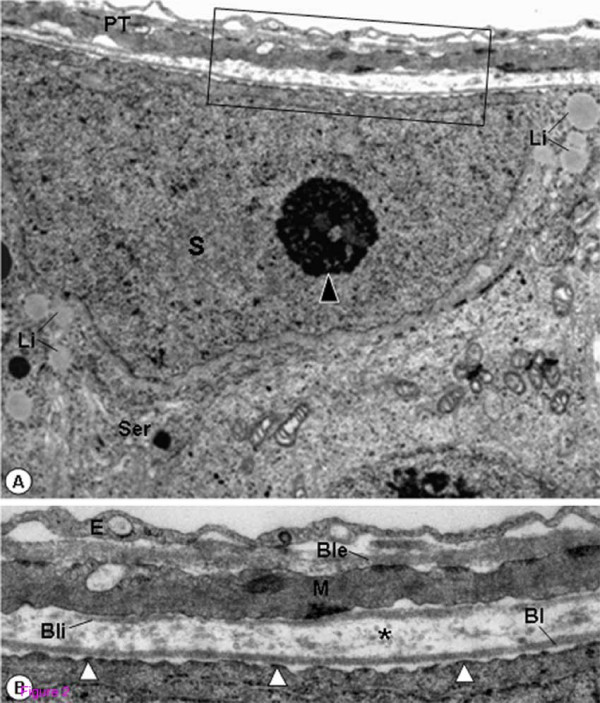
**Electron micrographs of portion of seminiferous tubule of rat from CG.** In **A**, the SC showing nucleus (S) with evident nucleolus (arrowhead), smooth endoplasmic reticulum (Ser) and lipid inclusions (Li), is adjacent to the peritubular tissue (PT). ×11,000. In **B **(high magnification of **A**), a well defined epithelial basal lamina (Bl) and a thin layer of type I collagen fibrils (asterisk) are observed. In the inner surface of the myoid cell (M), a discontinuous basal lamina is observed (Bli); the external surface is surrounded by a continuous basal lamina (Ble). Hemidesmosome-like junctions (white arrowheads) are anchoring SC to the lamina densa of the epithelial basal lamina. Endothelial cell (E). ×28,800.

In the tubules of rats from CG, the SC nuclei with evident nucleolus were located next to the basal lamina of the seminiferous epithelium. In the cytoplasm, mitochondria with tubular cristae, smooth endoplasmic reticulum and lipid inclusions were often observed. The peritubular tissue with rectilinear outline showed a thin layer of type I collagen fibrils between the basal lamina and the myoid cells. The inner and outer surfaces of the myoid cells were also surrounded by a discontinuous and continuous basal lamina, respectively. The surface of the Sertoli Cells plasma membrane was always in close proximity to the basement membrane; the hemidesmosome-like junctions were adhered to the lamina densa of the basal lamina (Figs. 2A and 2B).

**Figure 3:**
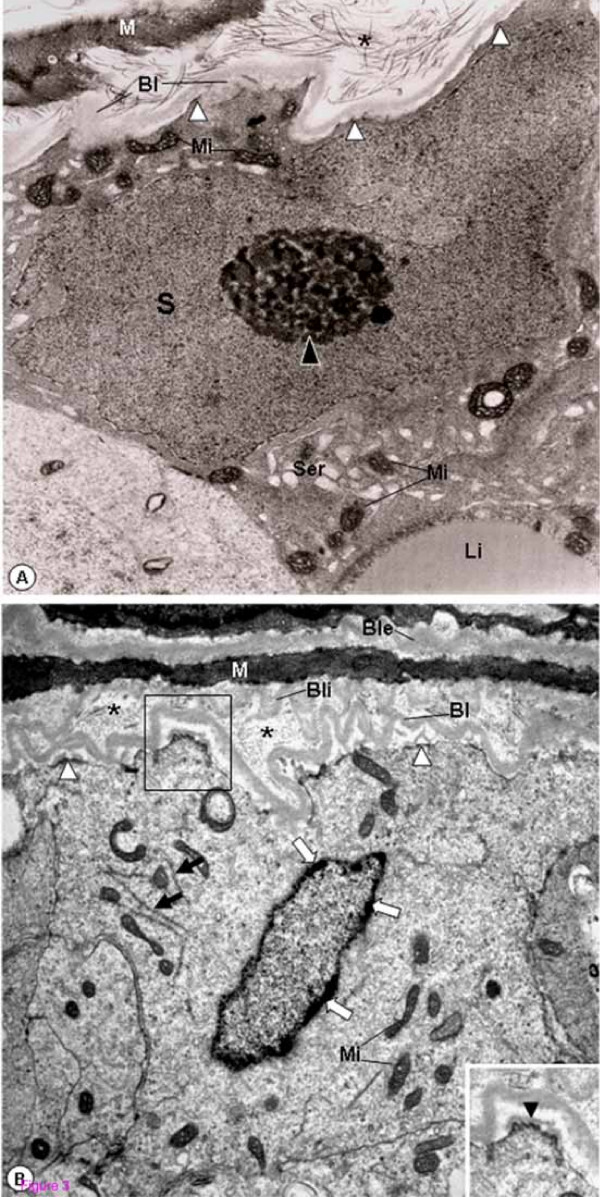
**Electron micrographs of portions of seminiferous tubules of rats from CmG. **In **A**, the irregular outlined tubule shows SC nucleus with normal aspect (S) and evident nucleolus (black arrowhead). Mitochondria with tubular cristae (Mi), smooth endoplasmic reticulum (Ser) and lipid inclusion (Li) are observed. Portions of the epithelial basal lamina are thickened (Bl). The layer between the myoid cell (M) and Bl is also thickened and shows numerous type I collagen fibrils (asterisk). The SC plasma membrane is adhered to the lamina densa by hemidesmosome-like junctions (white arrowheads). ×11,700. In **B**, the basal lamina (Bl) surrounding the irregularly outlined tubule is infolded. Adjacent to both inner and outer surfaces of the myoid cell (M), continuous and infolded basal laminae are observed (Bli and Ble). Between Bl and Bli, a thick layer with type I collagen fibrils (asterisks). Portions of the SC plasma membrane containing hemidemosome-like junctions are detached from the Bl (white arrowheads) and surrounded by a thin lamina of amorphous matrix (inset; arrowhead). In the SC, mitochondria (Mi) and filaments of the cytoskeleton (black arrows) are observed. In the nuclear periphery, electron dense clumps of chromatin are observed (white arrows). ×10,000. Inset: ×13,300.

**Figure 4:**
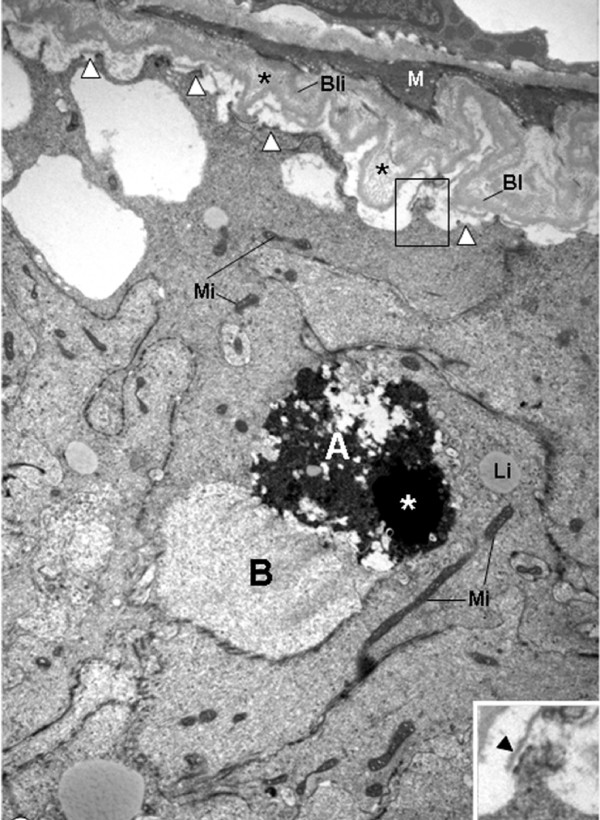
**Electron micrograph of portion of seminiferous tubule of rat from CmG. **The epithelial basal lamina (Bl) and the inner basal lamina (Bli) of myoid cell (M) are infolded. Between these laminae, a thick layer of type I collagen fibrils is observed (asterisks). In the SC plasma membrane, the hemidesmosome-like junctions are detached from the Bl (white arrowheads) and surrounded by a thin layer of amorphous matrix (inset; arrowhead). The SC nucleus is dislocated from the basal region and exhibits two portions: (A) an electron dense portion containing fragmented chromatin irregularly distributed and (B) an electron lucent portion with a thin granular chromatin homogeneously distributed. The portion "A" contains a block of condensed chromatin (white asterisk). Mitochondria with tubular cristae (Mi); lipid inclusions (Li). ×8,960; Inset: ×19,700.

**Figure 5:**
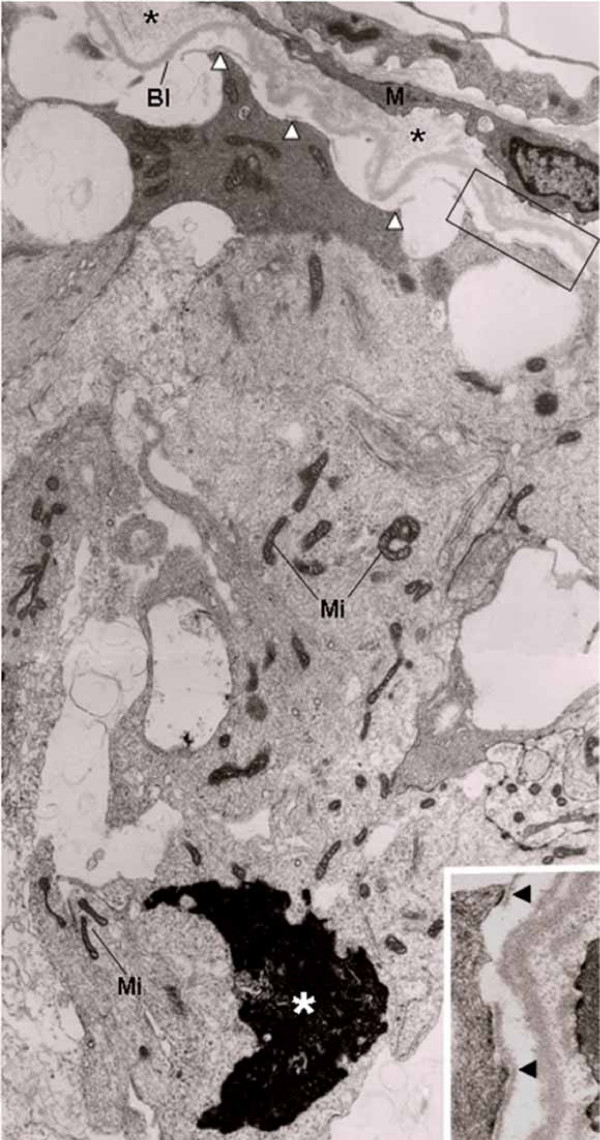
**Electron micrograph of portion of seminiferous tubule of rat from CmG.** The SC shows mitochondria with tubular cristae (Mi) and an irregular nuclear portion with condensed chromatin is observed in the apical portion (white asterisk). In all extension of the plasma membrane, the hemidesmosomes (white arrowheads) are detached from the thickened basal lamina (Bl) and surrounded by a thin lamina of amorphous matrix (inset; arrowheads). Between Bl and myoid cell (M), a thick layer of collagen fibrils is observed (asterisks). ×7,800; Inset: ×19,500.

In the seminiferous tubules of rats from CmG showing epithelium with normal aspect, the epithelial basal lamina was infolded, and a thickened layer of type I collagen fibrils between this basal lamina and the myoid cells was observed. However, the adjacent SC nuclei with evident nucleolus showed normal aspect. The plasma membrane was in close contact to the basement membrane and hemidesmosome-like junctions were often anchoring the SC to the basal lamina (Fig. 3A). In addition to these peritubular alterations, the basal lamina adjacent to the inner surface of myoid cells was also intensely infolded in some tubules. Adjacent to this damaged peritubular tissue, SC nuclei with condensed clumps of chromatin in the nuclear periphery were observed. In these cells, portions of the plasma membrane containing hemidemosome-like junctions were detached from the lamina densa and surrounded by a thin lamina of amorphous matrix (Fig. 3B). In tubules exhibiting similar peritubular alterations, the SC nucleus was dislocated from the basal portion and the nuclear chromatin was irregularly distributed in electron lucent portions containing a thin granular chromatin, and electron opaque portions with blocks of condensed chromatin (Fig. 4). In the apical portion of some Sertoli Cells, irregularly outlined blocks of condensed chromatin derived from nucleus were found. In these damaged Sertoli Cells, large extension of the plasma membrane containing hemidesmosome-like junctions was detached from the basal lamina (Fig. 5). The detached hemidesmosomes were usually surrounded by a thin layer of amorphous matrix (Figs. 4 and 5).

## Discussion

The significant reduction of peritubular tissue associated to the presence of apoptotic peritubular myoid cells adjacent to apparently normal epithelial areas have indicated that myoid cells are the primary cell affected by cimetidine treatment [3]. In the present study, the analysis of the damaged basement membrane-Sertoli Cells interface revealed that the alterations induced by cimetidine on the peritubular tissue may be responsible for the SC alterations, culminating in apoptosis and significant decrease in the number of these cells. These findings confirm a previous study in which a possible occurrence of cell death in the Sertoli Cells, evidenced by the TUNEL labelling, was suggested [5]. The TUNEL method alone is not considered a specific marker for apoptosis, but the morphological images (under light and electron microscopy) have been conclusive for identification of this classical type of cell death [20]. Additionally to the TUNEL-positive SC nuclei, our results showed the presence of nuclear changes and nuclear fragments of Sertoli Cells in the H&E and PAS stained sections. The ultrastructural images showed masses of chromatin in the SC nuclear periphery, condensed blocks of chromatin and chromatin disintegration, suggesting nuclear fragmentation – typical of apoptosis, as previously described in other cell types [20-23]. For the first time, typical images of Sertoli Cells undergoing apoptosis (from early to advanced stages) have been demonstrated "in vivo". The ultrastructural images illustrated in this study were obtained after the analysis of several ultrathin sections. Due to the thickness of the sections and low probability to find typical Sertoli cells associated to typical features of apoptosis, numerous Sertoli cells were analyzed for a long time.

Additionally to the apoptotic features, a high frequency of dislocated SC nuclei from their original basal site was observed in all treated animals. This alteration was associated to the presence of intraepithelial spaces, suggesting loss of contact between spermatids and Sertoli Cells. SC-germ cell attachment and positioning of the SC nucleus is maintained by vimentin – filaments of the cytoskeleton [24]. The collapse of the vimentin filaments around the nucleus seems to affect the integrity between Sertoli and germ cells [8,12]. Thus, our results also indicate a possible disruption of the SC cytoskeleton, which may be associated to apoptosis. This hypothesis is reinforced regarding the fact that some dislocated nuclei showed morphological aspects of apoptosis or were positive to the TUNEL method.

The functional and structural maintenance of Sertoli Cells, including the orientation of nuclear polarity are dependent on the SC-basement membrane interactions [25,26]. In the present study, a parallelism between the SC damage and changes in the basement membrane was strictly correlated. The lamina densa adjacent to the altered Sertoli Cells was usually thickened, infolded and, in some portions, detached from the SC plasma membrane. Thickening of basal lamina has been commonly observed in the testes under abnormal conditions [27,28]; however, morphological evidences that could explain the basal lamina enlargement are not found. The ultrastructural findings revealed a thin layer of amorphous lamina underlying the hemidesmosomes in the detached portions of the Sertoli Cells. Similar images regarding apoptotic or non-apoptotic SC-basement membrane interface as well as other epithelial cell types have not been demonstrated. However, similar findings are demonstrated in studies focusing the process of epidermal basal lamina formation which is initiated directly subjacent to hemidesmosomes [29,30] and, subsequently, continues to be formed under the inter-hemidesmosomal areas of the basal plasma membrane, forming a continuous lamina [30]. Therefore, the findings of the current study indicate a continuous production and deposition of lamina densa by the Sertoli Cells. Since duplication of lamina densa was observed surrounding the apparently healthy tubules [3], we propose that the neo-formed layers of lamina densa may be gradually fused with each other and with the original basal lamina, resulting in the thickened basal lamina. Therefore, detachment of Sertoli Cells and continuous and uncontrolled deposition of lamina densa are morphological evidences that contribute to the comprehension of the thickening of basal lamina during testicular disorders.

Why SC is not able to maintain the plasma membrane (hemidesmosomes) adhered to the basal lamina becomes questionable. Our findings strongly suggest a strict relationship between cellular detachment and apoptosis. In the Sertoli Cells exhibiting early signs of nuclear changes (early apoptotic process), only some anchoring sites – hemidesmosomes – were detached from the lamina densa. However, in the cells showing advanced nuclear apoptotic features, large portions of the plasma membrane were detached. The inhibition of the linkage between SC and laminin, by using anti-laminin IgG, have demonstrated disruption of the pericellular circumferal rings of F-actin filaments and subsequently focal detachment of SC [31]. Detachment of epithelial cells from matrix [32] and absence of the basement membrane for Sertoli cells [16] result in apoptosis. In corneal basement membrane, it has been proposed that laminin 5, the main component of lamina densa, plays a role in the transmission of extracellular survival signals, suppressing apoptosis [33]. Moreover, integrins, components of hemidesmosomes, are the main receptors for cell adhesion to extracellular matrix, and play critical roles in cytoskeletal dynamics, cell polarization, and cell migration [34]. In the testes, integrin α6β1 has been the main complex receptor mediating the adherence of SC to the basement membrane [35]. Studies have demonstrated that lack of functional β4 integrin leads to weak cellular adhesion and cell death by apoptosis in transgenic mice [36]. On the other hand, α6β4 integrin is cleaved by caspases-3 and -7 during apoptosis [37]. Thus, a possible disruption on the focal adhesion SC integrin-laminin may be related to detachment and apoptosis of SC. At least in part, these alterations may be associated to changes and/or death of myoid cells. Enlargement of the layer of collagen fibrils, normally produced by peritubular myoid cells [38], has been associated to altered myoid cells which produce tubular fibrosis [39]. Myoid cells secrete factors that stimulate SC activity [6] and express androgen receptors whose absence leads to impairment of the SC junctions [7]. Thus, we believe that the initial damage (structural or functional) in the SC may be derivative from alterations on the peritubular components, resulting in SC detachment and apoptosis. These alterations may be the main cause of germ cell loss by apoptosis and tubular atrophy in cimetidine-treated rats.

## Conclusion

The detachment of SC from the lamina densa coupled to the nuclear dislocation and apoptotic features strongly suggest that the process of apoptosis in SC is related to changes in the structural network of the cytoskeleton that maintain polarity and anchoring of these cells to their substratum. A possible interference on the myoid-Sertoli and/or matrix-SC paracrine interactions may be responsible for these alterations. The process of thickening of basal lamina, commonly observed under testicular abnormal or pathological conditions, may be focused regarding possible Sertoli cell detachment and continuous deposition of lamina densa by the injured Sertoli cells.

## Authors' contributions

ESC carried out the experiments, the tissue processing (staining and TUNEL method) and analyses under light microscopy and coordinated the study. PSC carried out the tissue processing and analysis under transmission electron microscopy; ESC and PSC examined and selected the ultrastructural images, participated in the design, writing and final revision of the manuscript.
